# Local Clays from China as Alternative Hemostatic Agents

**DOI:** 10.3390/molecules28237756

**Published:** 2023-11-24

**Authors:** Changjiao Gan, Hongjie Hu, Zhiyun Meng, Xiaoxia Zhu, Ruolan Gu, Zhuona Wu, Wenzhong Sun, Peng Han, Hongliang Wang, Guifang Dou, Hui Gan

**Affiliations:** 1 Department of Pharmaceutical Sciences, Beijing Institute of Radiation Medicine, Beijing 100850, China; gan10011020003@163.com (C.G.);; 2 National Medical Products Administration Institute of Executive Development, 16 Xi Zhan Nan Road, Beijing 100073, China; 3 Zhengzhou Institute of Multipurpose Utilization of Mineral Resources, Zhengzhou 450006, China

**Keywords:** clay, kaolin, halloysite, zeolite, hemostatic agent

## Abstract

In recent years, the coagulation properties of inorganic minerals such as kaolin and zeolite have been demonstrated. This study aimed to assess the hemostatic properties of three local clays from China: natural kaolin from Hainan, natural halloysite from Yunnan, and zeolite synthesized by our group. The physical and chemical properties, blood coagulation performance, and cell biocompatibility of the three materials were tested. The studied materials were characterized by using scanning electron microscopy (SEM), Fourier transform infrared spectroscopy (FTIR), X-ray diffraction (XRD), X-ray fluorescence spectroscopy (XRF), thermogravimetric analysis (TGA), and differential scanning calorimetry (DSC). All three clays showed different morphologies and particle size, and exhibited negative potentials between pH 6 and 8. The TGA and DSC curves for kaolin and halloysite were highly similar. Kaolin showed the highest water absorption capacity (approximately 93.8% ± 0.8%). All three clays were noncytotoxic toward L929 mouse fibroblasts. Kaolin and halloysite showed blood coagulation effects similar to that exhibited by zeolite, indicating that kaolin and halloysite are promising alternative hemostatic materials.

## 1. Introduction

Uncontrolled hemorrhage is a significant threat to life,. and it accounts for approximately 30% to 40% of traumatic deaths worldwide. Most fatalities on the battlefield can be attributed to severe hemorrhage within the first hour of receiving a wound. Achieving rapid effective hemostatic agents is essential in the global healthcare system, as they can significantly reduce the mortality rate caused by severe hemorrhage. The ideal hemostatic agents need to possess high efficiency, convenience, user-friendliness, stability, biocompatibility, cost-effectiveness, and eco-friendliness. However, achieving this goal still presents a significant challenge [1,2,3,4]. Aluminosilicate clays have been widely used in various fields, such as cosmetics, food, ceramics, and biomedicine, due to their abundant reserves, easy availability, good biocompatibility, inexpensiveness, and stability. Aluminosilicate clays may also be used as drug-delivery vehicles, promoting blood coagulation, and antibacterials [4,5,6,7,8,9], because of their lack of allergens derived from humans or animals, stable physical and chemical properties, as well as convenient production and transportation. Some studies have shown that zeolite [10,11], halloysite [12,13], and kaolin [14] are cost-effective and biocompatible minerals for this purpose. The prehydrated zeolite QuikClot ACS+ and kaolin-based hemostat QuikClot Combat Gauze are approved by the Food and Drug Administration (FDA) for external application because of their efficiency in hemorrhage control without the risk of thermal injury [15,16]. However, zeolites release a considerable amount of thermal energy when contacting blood; this exothermic reaction raises the wound temperature as high as 65 °C and can cause serious burns. Zeolites are no longer suitable for complex bleeding scenarios or stringent application conditions due to specific drawbacks [8,16,17].

Kaolin (kaolinite; Al_2_Si_2_O_5_(OH)_4_) is a hydrous aluminum phyllosilicate member belonging to the dioctahedral 1:1 kaolin mineral group. It comprises a silica tetrahedral layer (siloxane surface) covalently bonded to an alumina octahedral layer (aluminol surface) through an apical oxygen atom. Each kaolinite layer is considered as a strong dipole and the siloxane surface exhibits negative charges and is highly hydrophobic, whereas the aluminol surface is positively charged and is hydrophilic [18,19,20]. Halloysite (Al_2_Si_2_O_5_(OH)_4_·2H_2_O) is a naturally occurring aluminosilicate with a 1:1 ratio between alumina and silica layers, also belonging to the kaolin mineral group, which structurally comprises ultramicroscopic multilayered hollow cylinders [21,22]. Halloysites are commonly known as halloysite nanotubes (HNTs) as a mesoporous substance due to their nanostructure, with a specific surface area ranging from 50–60 m^2^/g and a bulk density of approximately 2.53 g/cm^3^ [9]. Some HNTs exhibit a spherical morphology, whereas others possess an extremely elongated structure that can extend up to several microns in length [9]. The inner surface comprises aluminum hydroxide, and the external surface of silicon dioxide confers a positively charged inner lumen [2]. They can be considered a potential alternative to conventional minerals [23,24] because they are biocompatible [1,25], easily dispersed in a polymer matrix, and form suspensions that are stable for 2–3 h [26,27]. The structural differences between kaolin and halloysite are caused by the distribution of vacant sites in the octahedral sheet, the stacking interlayer expansion, and the hydroxyl group orientations [20]. Kaolin and halloysite are aluminosilicate clays that may promote coagulation by activating the Hageman factor (factor XII) of the intrinsic pathway without an exothermic reaction [7,15,28].

Zeolites are a family of microporous crystalline aluminosilicate minerals with a network of 0.3–1.5 nm-wide pores [29]; zeolites can stop bleeding, balance the body pH, stimulate skin wound regeneration, and neutralize and eliminate noxious substances (toxins, heavy metals, ammonium, and nitrosamines) [30]. Their framework contains silicon, aluminum, and oxygen, whereas the pores contain cations, water, and other molecules [30,31] that may interact with the zeolites through dipolar interactions. There are over 80 different types of zeolites in nature and with more than 247 recognized structures in International Zeolite Association (IZA) database [32,33]. Zeolites exchange cations such as Na^+^, K^+^, Ca^2+^, and Mg^2+^ through chemical–physical interactions, and the exchange capacity mainly depends on the silicon-to-aluminum ratio [31]. Zeolites can be synthesized from alkaline-assisted preactivated halloysite [34] and kaolin [35] via different methods [32]. Zeolite A can be used as an effective hemostat [36,37].

The objective of this study was to assess the blood coagulation properties of two local natural clays, kaolin and halloysite, obtained from two different locations in China. The two materials showed a blood coagulation performance similar to that of synthetic zeolite, suggesting that they are promising alternative hemostatic materials.

## 2. Results

### 2.1. Characterization

**Figure 1:**
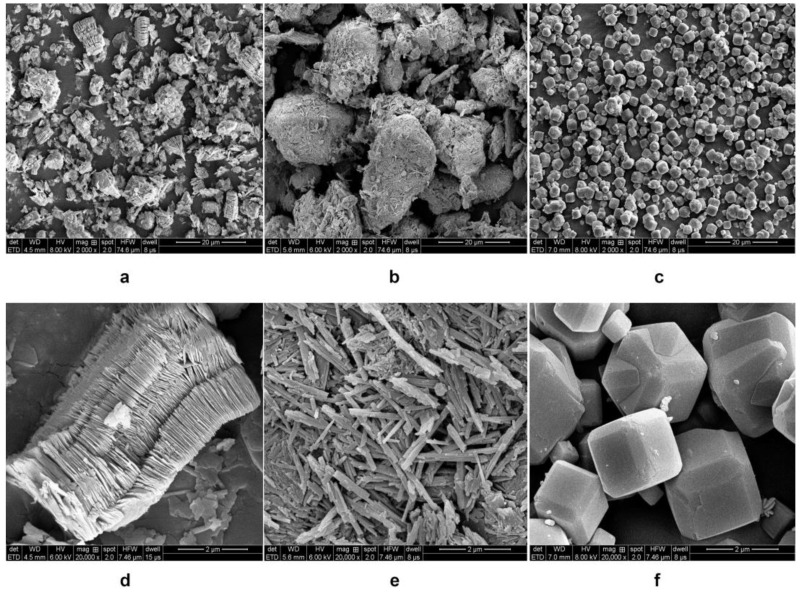
SEM images of (**a**,**d**) kaolin, (**b**,**e**) halloysite, and (**c**,**f**) zeolite. Magnification 2000× (**a**–**c**) and 20,000× (**d**–**f**). Scale bar: (**a**–**c**) 20 μm and (**d**–**f**) 2 μm.

The morphology of kaolin, halloysite, and zeolite was studied using SEM (Figure 1). Kaolin shows the booklet and stacked layered structure composed of typical pseudohexagonal particles with a size of 0.5–2.0 µm (Figure 1a,d). Halloysite exhibits a tubular morphology with smooth, clear-edged, and hollow lumens (Figure 1b,e) [26,38]. Zeolite shows cubic particles with smooth surfaces and a size of approximately 0.5–2.0 µm (Figure 1c,f). Zeolite exhibits a significantly higher Brunauer–Emmett–Teller (BET) surface area (747.17 m^2^/g), nearly 32 times larger than that of kaolin (23.43 m^2^/g) and 13 times larger than that of halloysite (57.3 m^2^/g). At the same time, the pore diameter of zeolite (1.63 nm) is considerably smaller than those of kaolin (14.68 nm) and halloysite (15.93 nm).

**Figure 2:**
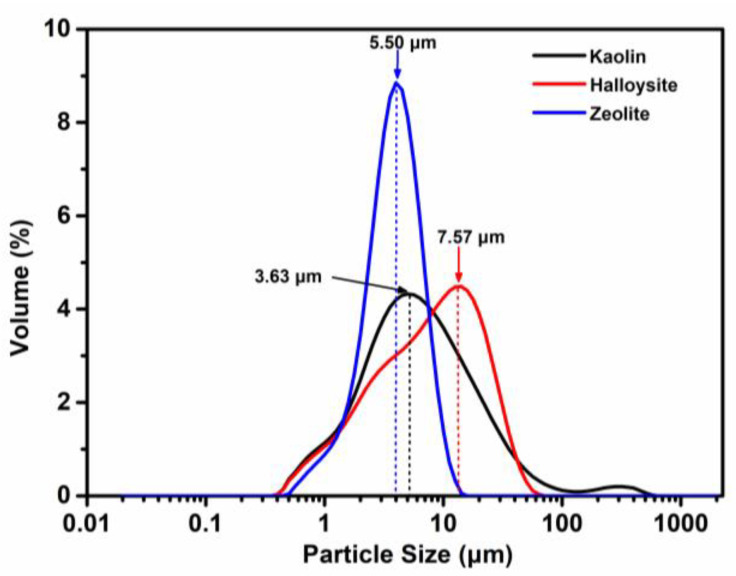
Particle size distributions of kaolin, halloysite, and zeolite.

The particle size distributions of kaolin, halloysite, and zeolite are shown in Figure 2. The particle size of kaolin ranges from 0.40 to 563.68 μm, with most particles < 100 μm, presenting a much wider size distribution than zeolite (0.45–14.16 μm) and halloysite (0.40–63.24 μm). The average particle sizes (D_0.5_) of kaolin, halloysite, and zeolite are 3.63 μm, 7.57 μm, and 5.50 μm, respectively.

**Figure 3:**
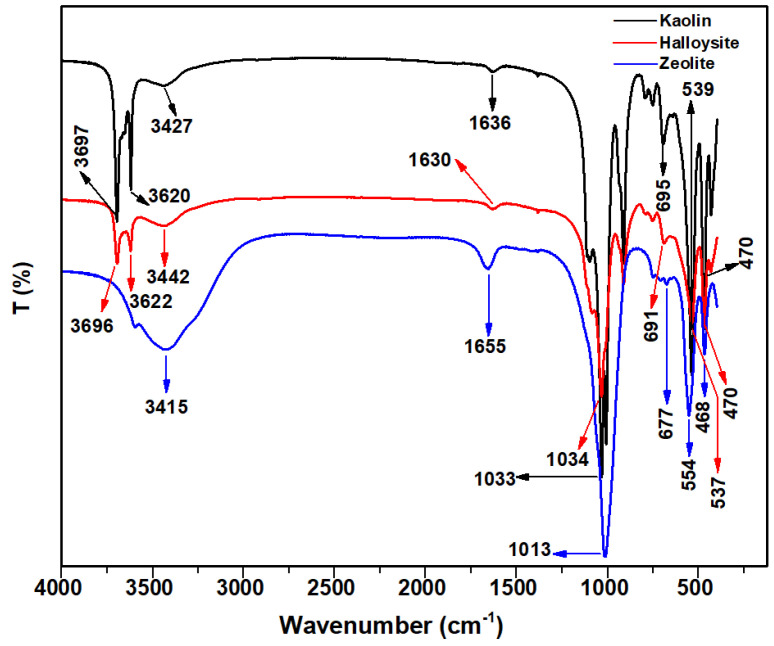
FTIR spectra of kaolin, halloysite, and zeolite.

The FTIR spectra of kaolin, halloysite, and zeolite are presented in Figure 3. For halloysite and kaolin, the band located at approximately 3700 cm^−1^ represents the stretching vibration of the inner surface O–H groups, and the band at approximately 3620 cm^−1^ represents the stretching vibration of the inner groups. The inner surface O–H groups are connected to the Al–centered octahedral sheets and form hydrogen bonds with the oxygen sheet in the next double layer [26]. The band at approximately 1630 cm^−1^ was assigned to the bending vibration of the O–H groups. The band at approximately 1030 cm^−1^ was ascribed to the stretching vibration of the Si–O and Al–O groups. The peak band at approximately 470 cm^−1^ was attributed to the bending vibration of the Si–O–Al groups in the structure. The peaks at 677 cm^−1^ and 554 cm^−1^ in zeolite were ascribed to the vibration modes of its framework [39]. The bands at 3415 cm^−1^, 1655 cm^−1^, and 1013 cm^−1^ exhibit greater intensity in zeolite than in halloysite and kaolin. Additionally, the broad band at approximately 3414 cm^−1^ corresponds to the stretching vibration of the adsorbed water and inner surface O–H groups.

The XRF results are presented in Table 1. The major chemical compounds of the minerals are SiO_2_ and Al_2_O_3_ and the compositions of the three clays are similar. The CaO content in zeolite is considerably higher than that in halloysite and kaolin. At the same time, the contents of K_2_O and Fe_2_O_3_ in halloysite and kaolin are higher than those in zeolite.

**Figure 4:**
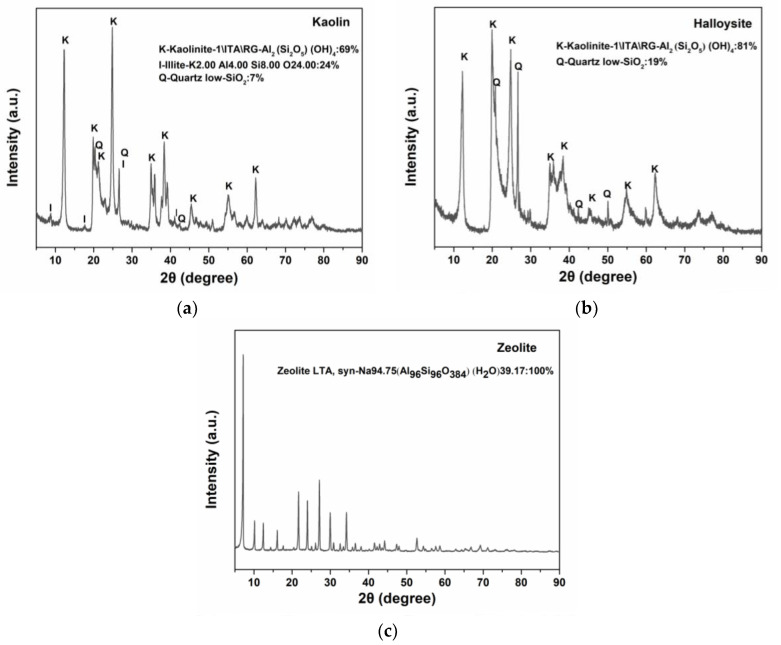
XRD patterns of (**a**) kaolin, (**b**) halloysite, and (**c**) zeolite.

Figure 4 shows the XRD patterns of the clay powders in the range of 2θ = 5°–90°. In the XRD patterns of kaolin and halloysite, kaolinite exhibits strong reflections, with a few reflections attributed to quartz. Calculations based on the Inorganic Crystal Structure Database (ICSD) [40] data cards of the minerals show that the weight fraction of kaolinite in kaolin is 69 wt.%, illite is 24 wt.%, and quartz 7 wt.%; at the same time, the weight fraction of kaolinite in halloysite is 81 wt.%, and that of quartz is 19 wt.%. XRD analysis shows that both kaolin and halloysite are typical kaolinites. For zeolite, sharp peaks in the XRD pattern indicate high crystallinity; furthermore, the observed peaks comply with the reference values for Linde Type A (LTA) zeolites according to JCPDS 01-089-8015.

**Figure 5:**
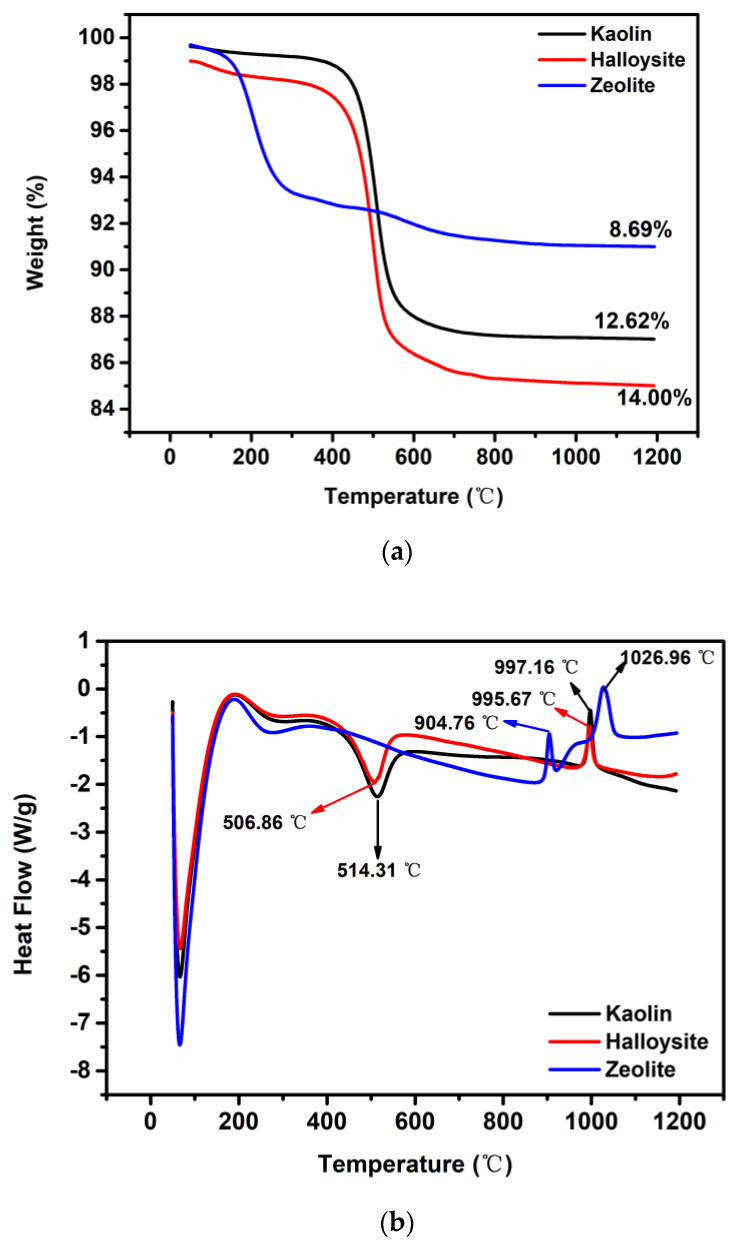
(**a**) TGA and (**b**) DSC curves of kaolin, halloysite, and zeolite.

The TGA and DSC results confirm the thermal stability of kaolin, halloysite, and zeolite. The TGA and DSC curves (Figure 5a,b) for kaolin and halloysite are highly similar. The major mass loss in kaolin and halloysite occurs at 400–600 °C (Figure 5a), and only one endothermic peak is observed at approximately 500 °C [41] (Figure 5b), which was attributed to the dihydroxylation [42,43] of the interlayer water bound to the clay minerals and the removal of other volatile materials [23,26,44]. Most of the impurities in the clays volatize at <600 °C; therefore, the TGA curves above this temperature are flat. In addition, the exothermic peaks observed at 995.67 °C (halloysite) and 997.16 °C (kaolin) were attributed to the crystallization of mullite [45]. The residual mass of zeolite at 1200 °C is higher than those of kaolin and halloysite. The major mass loss in zeolite occurs at 50–250 °C, whereas kaolin and halloysite are relatively stable up to 400 °C. The major broad endothermic peaks at <800 °C in the DSC traces of zeolite (Figure 5b) correspond to framework dehydration. The structure of zeolite collapses at >800 °C [46].

### 2.2. Zeta Potentials

**Figure 6:**
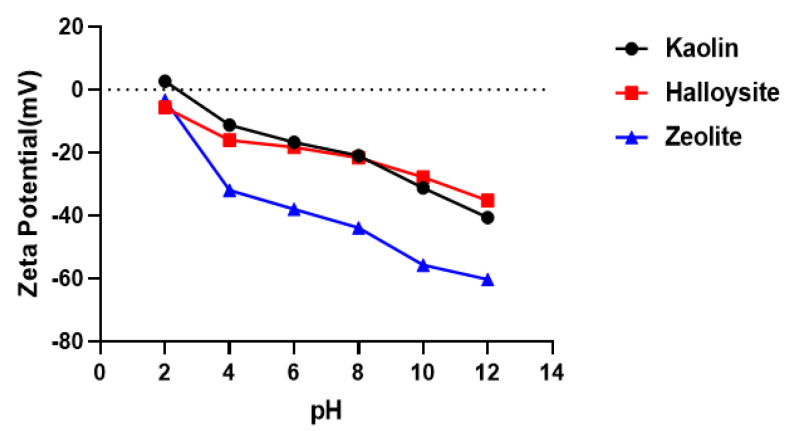
Zeta potentials of kaolin, halloysite, and zeolite depending on the pH. The dash line means Zeta Potential “0 mV”.

To investigate the effects of surface charge on contact activation of the coagulation cascade, we measured the zeta potentials of kaolin, halloysite, and zeolite in the pH range of 2–12 (Figure 6). The zeta potential between pH 6–8 of zeolite is −38.0 ± 0.7 to −43.9 ± 0.4 mV, that of kaolin is −16.7 ± 1.1 to −21.0 ± 1.2 mV, and that of halloysite is −18.3 ± 0.9 to −21.6 ± 0.4 mV. The surface charge at pH 6–8 deserves special attention because blood has a pH of 7.35–7.45. All samples exhibit negative potentials between pH 6 and 8; their zeta potentials become more negative with increasing pH. Zeolite shows a more negative surface charge than kaolin and halloysite in the pH range of 6–8.

### 2.3. Water Absorption

**Figure 7:**
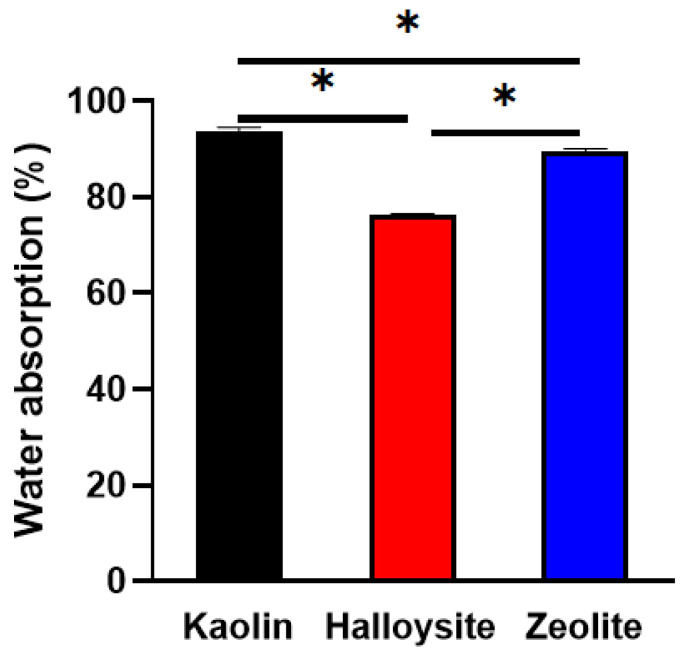
Water absorption properties of kaolin, halloysite, and zeolite (*n* = 3, * < 0.05).

The mass of the clay increases as it absorbs water through the porous capillaries. The results of the water absorption experiments are presented in Figure 7. Kaolin shows the highest water absorption capacity (approximately 93.8% ± 0.8%), followed by halloysite (76.3% ± 0.3%) and zeolite (89.5% ± 0.5%).

### 2.4. Cytotoxicity Studies

**Figure 8:**
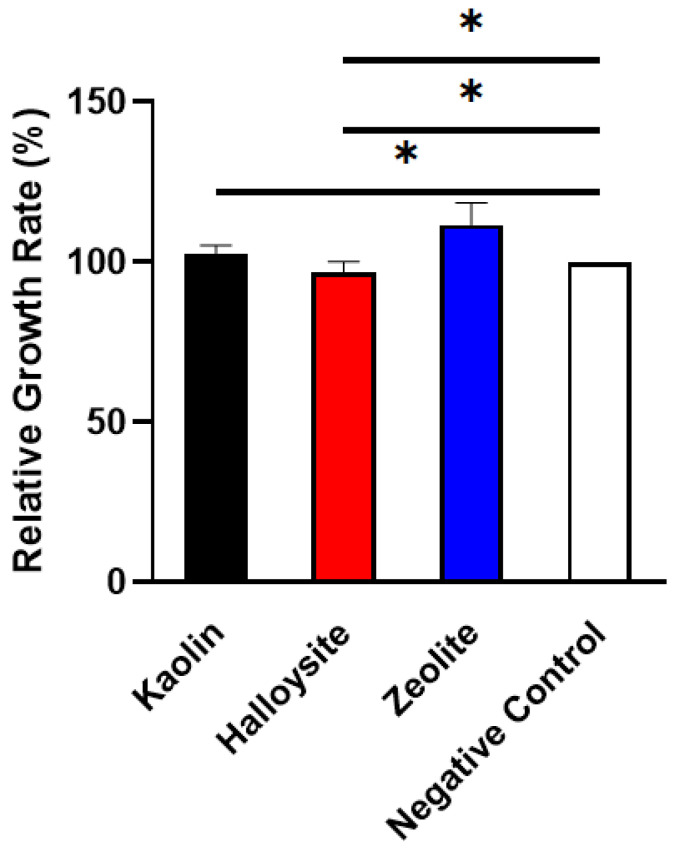
Cytotoxicity of kaolin, halloysite, and zeolite using MTT assay (*n* = 3, * < 0.05).

An ideal biomaterial should have low cytotoxicity and high biocompatibility. Therefore, the cytotoxicity of kaolin, halloysite, and zeolite was assessed using the MTT assay. The relative growth rate (RGR) of L929 with the clay extractants after culturing for 48 h is shown in Figure 8. All three clays are noncytotoxic and biocompatible with L929 fibroblast cells. The results indicate that the investigated inorganic minerals may be used as biomedical raw materials.

### 2.5. Plasma Clotting Assay

**Figure 9:**
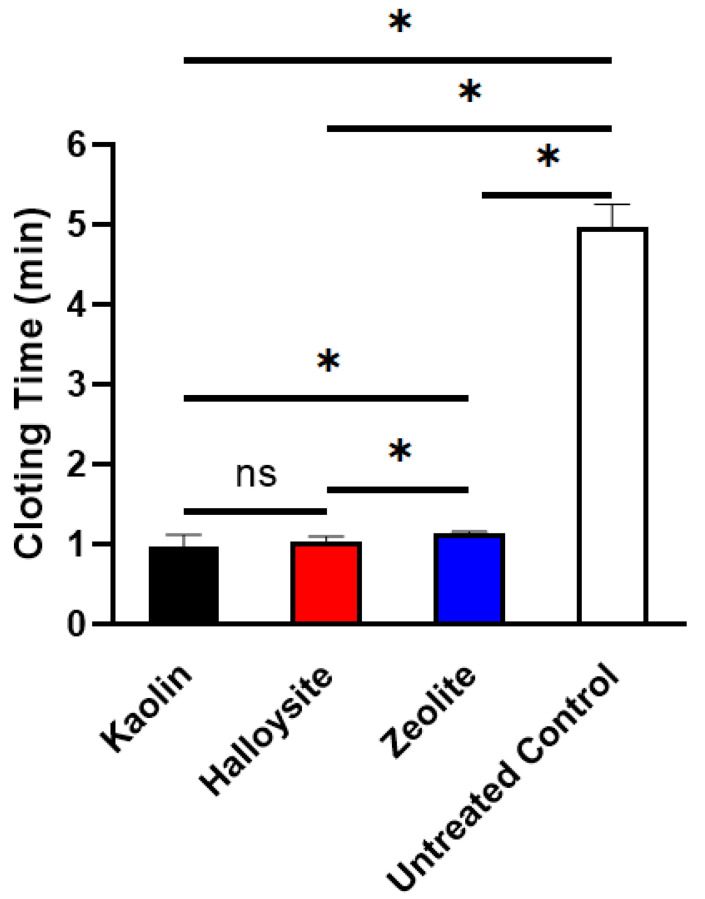
PRTs of the three minerals (*n* = 6, * < 0.05, ns: no statistical difference, “ns” means no statistical difference).

Plasma recalcification time (PRT) is an important indicator of endogenous coagulation cascade activation, indicating the time required for fibrin clot formation when calcium is replenished in the anticoagulated plasma [32,47]. The data in Figure 9 show that the PRTs of kaolin (0.98 ± 0.13 min) and halloysite (1.04 ± 0.05 min) are shorter than those of zeolite (1.14 ± 0.03 min, *p* < 0.05). At the same time, the PRT of halloysite was not significantly different from that of kaolin (*p* > 0.05). Notably, the PRTs of all clays were shorter than those of the untreated control (4.98 ± 0.28 min) in our previous study [48]. The procoagulant activity and stability of the clays were further confirmed through the PRT assay, demonstrating the superiority of halloysite and kaolin over zeolite.

### 2.6. In Vitro Blood Clotting Test

**Figure 10:**
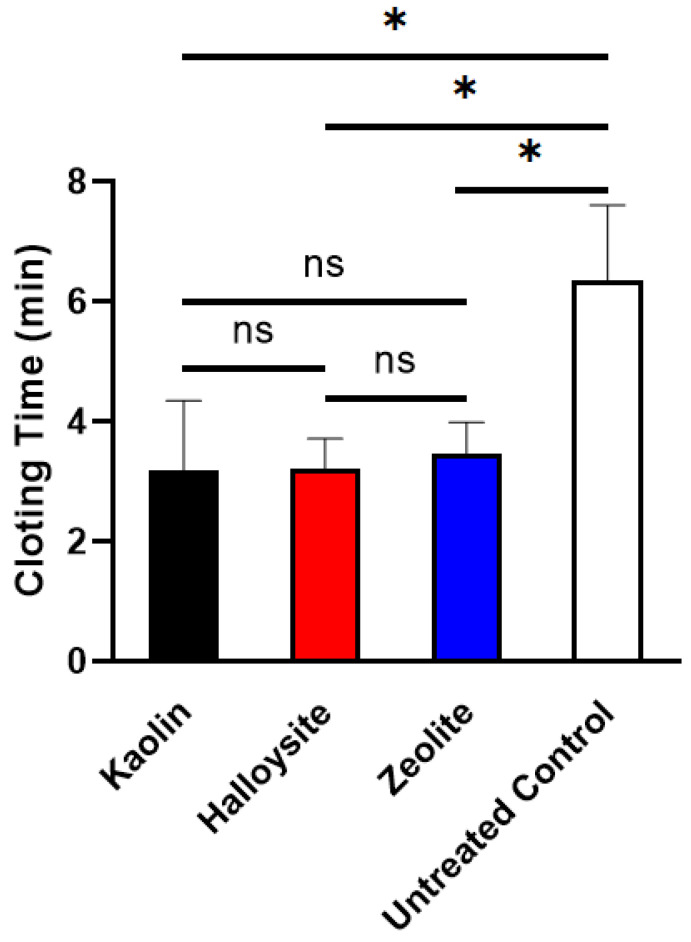
Blood clotting times of kaolin, halloysite, and zeolite (*n* = 5, * < 0.05, ns: no statistical difference, “ns” means no statistical difference).

Blood clotting time directly reflects the procoagulant activity. As shown in Figure 10, no significant differences (*p* > 0.05) were observed among the average clotting times for kaolin (3.19 ± 1.15 min), halloysite (3.21 ± 0.51 min), and zeolite (3.47 ± 0.51 min). All of these samples showed shorter average clotting times than the untreated control (6.89 ± 0.58 min) in our previous study [48]. It is well known that phyllosilicate clays with negatively charged surfaces (such as kaolin and halloysite) can rapidly convert FXII to its active form (FXIIa), causing coagulation through the intrinsic pathway [15,49,50].

## 3. Discussion

Herein, three minerals were characterized and assessed. The physicochemical and thermogravimetric analyses demonstrated the coagulation-promoting surface chemistry and porous structure of the minerals. Halloysite and kaolin are more effective for blood coagulation than zeolite, suggesting that they are promising hemostatic materials. The SEM images showed that kaolin and halloysite have different morphologies and particle diameters. Microscopically, kaolin has a booklet and stacked layered structure and appears as stacked pseudohexagonal particles, whereas halloysite exhibits a tubular morphology with smooth, clear-edged, and hollow lumens. The tubular structure and high aspect ratio of halloysite provide a higher surface area and pore volume than the multilayer stacked structure of kaolin [8]. Fe_2_O_3_ in halloysite (1.17 wt.%) is higher than in kaolin (0.99 wt.%). It has been proven that the larger Fe^3+^ replaced Al^3+^ and the interlayer water molecules are housed within the halloysite structure [20]. A biocompatibility analysis revealed that the minerals are noncytotoxic toward L929 mouse fibroblasts. The content of CaO in zeolite is significantly higher than in halloysite and kaolin. Ca^2+^ (known as clotting factor IV) is a key factor in promoting blood coagulation because it serves as the ionic bridge between two negatively charged residues, such as cellular surface and clotting factors. The Ca^2+^ contained in zeolite would exchange with the Na^+^ and K^+^ in blood because cation exchange is a fundamental chemical property of zeolite. Moreover, Ca^2+^ could absorb the water molecules in the blood through an electrostatic interaction to concentrate the platelets and clotting factors, accelerating blood coagulation [17,19]. The content of Fe_2_O_3_ in halloysite and kaolin is significantly higher than in zeolite. Fe_2_O_3_ can facilitate RBC aggregation and clotting [18], which may be an important factor for blood clotting activation by kaolin and halloysite. The surface charge and isoelectric point of inorganic oxides promote coagulation through the contact activation pathway. Basic oxides with an isoelectric point above the pH of blood are anticoagulant, whereas acidic oxides with an isoelectric point below the pH of blood are procoagulant [51]. It is, thus, speculated that different surface morphologies of the clays result in different effects on blood. Major changes in the morphology of zeolite after modification confirm the XRD results, as previously described. The average particle sizes (*D*_0.5_) of kaolin, halloysite, and zeolite are 3.63, 7.57, and 5.50 μm, respectively. Halloysite has the largest particle size, but not the shortest PRT, indicating that particle size might not be the critical factor in the hemostatic performance of the clays.

The three clays exhibited both common and individual features, which is influenced by their diverse structures and physicochemical properties. It is well known that the hemostatic effects of clays were mainly attributed to three aspects: (i) the water absorption of the hydrophilic surface; (ii) the contact activation of the intrinsic coagulation pathway; and (iii) the formation of physical barriers through adhesion to tissue [8]. The capacity to absorb liquid, interaction with blood cells, and activation of the coagulation cascade affect the activity of a hemostatic agent [52]. Herein, kaolin showed the highest water absorption capacity (93.8% ± 0.8%) despite having the lowest specific surface area (23.43 m^2^/g). The efficient water adsorption by kaolin is attributed to its booklet and stacked layered structure. Additionally, the negatively charged surfaces of the clays may have promoted the activation of the intrinsic blood coagulation pathway [50]. The intrinsic blood clotting pathway is propagated downstream by factor XIa, also termed the plasma kallikrein/kinin system, causing thrombin formation. Factor XII is a substrate for kallikrein; the activated factor XII on the surface converts factor XI and prekallikrein to their activated forms (factor XIa and kallikrein, respectively), enabling rapid activation of the intrinsic pathway [53]. Kaolin and halloysite promote the activation of factor XII (Hageman factor) in the presence of prekallikrein and high-molecular-weight kininogen [54]. Platelet aggregation results in the formation of an embolus that promotes blood clotting [55]. Zeolite can quickly adsorb water from blood through concentrating natural clotting elements at the site of bleeding [16]. The highly porous surface and pore size are important factors for Ca-zeolite as an adsorbent to provide a higher surface area for efficient uptake [30]. The studied clays achieved rapid hemostasis through efficient water absorption, platelet concentration, clotting factors, and charge stimulation. At the same time, the negatively charged surface of the clays stimulated the conversion of FXII to FXIIa and activated the intrinsic coagulation pathway to generate thrombin, leading to fibrin formation [4,15,49,50]. Therefore, these inorganic hemostatic agents can prevent massive blood loss, facilitating and accelerating hemostasis. Future studies will be conducted on animal models of hemorrhage to obtain further scientific evidence for using inorganic composites for bleed control.

In conclusion, we found that among the three examined clays, kaolin showed the shortest bleeding time and the highest water absorption capacity. Halloysite is more suitable for drug delivery than kaolin and zeolite because of its tubular morphology and pore diameter. The three investigated clays exhibited excellent biocompatibility and hemostatic activity. Our results indicate that kaolin from Hainan, halloysite from Yunnan, and zeolite synthesized by our group are effective locally sourced hemostatic agents. Clay-based hemostatic agents show great promise for a safe and effective alternative to traditional rapid hemostasis.

## 4. Methods

### 4.1. Materials

The clay minerals used in this study were provided by the Zhengzhou Institute of Multipurpose Utilization of Mineral Resources. Kaolin and halloysite, collected from the Hainan and Yunnan provinces in China, respectively, were ground into powders. Both kaolin and halloysite powders were treated by cyclone and hydraulic classification, and feldspar and quartz sand were then sieved out. Finally, the two powders were dried for 2 h at 300 °C and screened through a 100-mesh sieve. The Ca-exchanged zeolite with a calcium ion exchange degree of 75% was obtained from the Zhengzhou Institute of Multipurpose Utilization of Mineral Resources. The zeolite was dried for 1 h at 500 °C and screened through a 100-mesh sieve. Particles below 150 μm were selected for further analysis. All animal experiments were performed with the permission of the Institutional Animal Care and Use Committee (IACUC) of the Laboratory Animal Center; the ethical approval number was IACUC of AMMS-13-2016-017 [48].

### 4.2. Characterization

SEM (Quanta FEG 250, Hillsboro, OR, USA) was used to observe the morphologies of halloysite, kaolin, and zeolite. The clays were sputter-coated with platinum before observation.

The particle size distribution was analyzed using a Malvern Mastersizer 2000 (Malvern Instruments Ltd., Malvern, UK). All samples were sonicated for 30 min before measurement.

A Micrometrics ASAP 2460 (Norcross, GA, USA) instrument was employed to perform the BET experiments. FTIR of the samples was performed using Nicolet 6700 (Thermo Fisher Scientific, Waltham, MA, USA).

The chemical composition of the samples was determined using XRF spectroscopy (ARL ADVANT XP+, Thermo Fisher Scientific, Waltham, MA, USA).

XRD of the samples was performed on an Xpert Pro MPD diffractometer (Malvern Panalytica, Eindhoven, Netherlands), with scanning conducted in the 2θ range of 5–90°.

TGA and DSC were performed using an SDT Q600 (TA Instruments, New Castle, DE, USA). All samples were heated from 50 °C to 1200 °C at a heating rate of 10 °C min^−1^ under a nitrogen atmosphere.

### 4.3. Zeta-Potential

The zeta potentials in the pH range of 2–12 of kaolin, halloysite, and zeolite were measured using a Malvern Zetasizer Nano ZS (Malvern Instruments Ltd., Malvern, UK). The samples were dispersed in deionized water at a concentration of 0.1 mg/mL and sonicated for 3 min before analysis.

### 4.4. Water Absorption

Water absorption was determined according to the Chinese national standard GB/T 20973-2007 [56]. The weight was measured for 2 h at 20 °C and 0.1 MPa; the water absorption rate (*W*_a_) was calculated using Equation (1):

*W*_a_ = (*W* − *W*_0_ − *m*)/*m* × 100, (1)

where *W*_a_ is the water absorption rate (%), *W* is the weight of wet filter paper and clay after water absorption (g), *W*_0_ is the weight of wet filter paper (g), and *m* is the weight of the clay before water absorption. The experiment was repeated 3 times, and an average value was obtained.

### 4.5. Cytotoxicity Study

The samples were sterilized via Co_60_ gamma irradiation at a dose of 25 kGy and incubated at 37 °C for 24 h. Subsequently, the extractant was separated via centrifugation. L929 mouse fibroblast cells (Cell bank of Chinese Academy of Sciences) were spread in a 96-well plate (5 × 10^3^/well) and cultured in RPMI 1640 medium (10% *v*/*v* fetal bovine serum, 1% *v*/*v* penicillin–streptomycin) at 37 °C for 24 h. Subsequently, the RPMI 1640 medium was replaced with the extractants from the clay samples, and the cells were incubated for an additional 48 h. The extractants were removed and dimethylsulfoxide (150 mL/well) was added to the wells after exposure to the MTT solution for 4 h. The absorbance of the formazan solution at 490 nm was measured using a SpectraMax 190 (Molecular Devices, San Jose, CA, USA). The control included cells in RPMI 1640 medium and cells in RPMI 1640 medium supplemented with 0.30% phenol. Cell viability was assessed using the relative growth rate (RGR), which was calculated using Equation (2):

RGR (%) = *A*_sample_/*A*_negative_ × 100%, (2)

where *A*_sample_ is the absorbance of the sample and *A*_negative_ is the negative control [32,57].

### 4.6. Plasma Clotting Assay

Fresh blood from New Zealand rabbits was collected and immediately mixed with 3.8% sodium citrate at a volume ratio of 9:1. Anticoagulated blood was then centrifuged at 3000 rpm for 15 min to obtain platelet-poor plasma. Subsequently, 0.1 mL platelet-poor plasma was quickly mixed with 0.1 mL of the assessed clay suspension (1 mg/mL). Each tube was incubated at 37 °C for 3 min, followed by the addition of 0.1 mL CaCl_2_ solution to start the plasma recalcification measurement [17,48]. The thrombus formation time was recorded (*n* = 6), and the values are reported as mean ± SD.

### 4.7. In Vitro Blood Clotting Test

The blood coagulation activity tests of clays were performed using fresh whole blood from a beagle dog. Briefly, 1 mL fresh whole blood was slowly added into flat-bottomed glass vials containing 5 mg clay samples (preheated to 37 °C) and gently inverted for 3 s. The time was recorded immediately after the addition of blood, and the vials were tilted every 10 s to determine if coagulation occurred. Five parallel tests were performed, and the results are reported as mean ± SD.

### 4.8. Statistical Analysis

Data were analyzed using ANOVA, using SAS statistical software (version 9.1, SAS Institute Inc., Cary, NC, USA) or version 8.0.1, GraphPad Prism. Data are expressed as mean ± SD; *p* < 0.05 was considered statistically significant.

## Figures and Tables

**Table 1:** Chemical compositions of the studied clays (wt.%).

Oxides	SiO_2_	Al_2_O_3_	K_2_O	Fe_2_O_3_	TiO_2_	Na_2_O	CaO	MgO	ZnO	^a^ LOI
Kaolin	47.23	36.03	1.65	0.99	0.44	0.496	0.02	0.03	0.00	12.59
Halloysite	46.34	35.59	0.38	1.17	0.15	0.08	0.866	0.11	0.05	14.73
Zeolite	38.08	31.06	0.09	0.07	0.01	2.30	20.62	0.18	0.00	7.43

^a^ LOI: loss on ignition at 980 °C.

## Data Availability

All data are available upon request from the corresponding author.
